# Mutability and Importance of a Hypermutable Cell Subpopulation that Produces Stress-Induced Mutants in *Escherichia coli*

**DOI:** 10.1371/journal.pgen.1000208

**Published:** 2008-10-03

**Authors:** Caleb Gonzalez, Lilach Hadany, Rebecca G. Ponder, Mellanie Price, P. J. Hastings, Susan M. Rosenberg

**Affiliations:** 1 Department of Molecular and Human Genetics, Baylor College of Medicine, Houston, Texas, United States of America; 2 Interdepartmental Graduate Program in Cell and Molecular Biology, Baylor College of Medicine, Houston, Texas, United States of America; 3 Department of Biology, University of Iowa, Iowa City, Iowa, United States of America; 4 Department of Molecular Virology and Microbiology, Baylor College of Medicine, Houston, Texas, United States of America; 5 Dan L. Duncan Cancer Center, Baylor College of Medicine, Houston, Texas, United States of America; 6 Department of Biochemistry and Molecular Biology, Baylor College of Medicine, Houston, Texas, United States of America; Université Paris Descartes, INSERM U571, France

## Abstract

In bacterial, yeast, and human cells, stress-induced mutation mechanisms are induced in growth-limiting environments and produce non-adaptive and adaptive mutations. These mechanisms may accelerate evolution specifically when cells are maladapted to their environments, i.e., when they are are stressed. One mechanism of stress-induced mutagenesis in *Escherichia coli* occurs by error-prone DNA double-strand break (DSB) repair. This mechanism was linked previously to a differentiated subpopulation of cells with a transiently elevated mutation rate, a hypermutable cell subpopulation (HMS). The HMS could be important, producing essentially all stress-induced mutants. Alternatively, the HMS was proposed to produce only a minority of stress-induced mutants, i.e., it was proposed to be peripheral. We characterize three aspects of the HMS. First, using improved mutation-detection methods, we estimate the number of mutations per genome of HMS-derived cells and find that it is compatible with fitness after the HMS state. This implies that these mutants are not necessarily an evolutionary dead end, and could contribute to adaptive evolution. Second, we show that stress-induced Lac^+^ mutants, with and without evidence of descent from the HMS, have similar Lac^+^ mutation sequences. This provides evidence that HMS-descended and most stress-induced mutants form via a common mechanism. Third, mutation-stimulating DSBs introduced via I-SceI endonuclease in vivo do not promote Lac^+^ mutation independently of the HMS. This and the previous finding support the hypothesis that the HMS underlies most stress-induced mutants, not just a minority of them, i.e., it is important. We consider a model in which HMS differentiation is controlled by stress responses. Differentiation of an HMS potentially limits the risks of mutagenesis in cell clones.

## Author Summary

Mutational processes are being discovered in which bacterial, yeast, and human cells under various stresses activate programs that increase mutagenesis, often under the control of cellular stress responses. These programs may potentially increase genetic variability in populations specifically when they are maladapted to their environments, i.e., when they are stressed. When mutation supply is limiting for evolution (for example, in small populations), these mechanisms might enhance the intrinsic ability of organisms/cells/populations to evolve, specifically during stress. Stress-induced mutagenesis mechanisms recast understanding of, and strategies for combating, problems such as host-pathogen interactions, generation of bacterial antibiotic resistance, cancer progression, and evolution of chemotherapy resistance, all problems of evolution of fitter variant clones fueled by genetic change under stress. A key problem in stress-induced mutagenesis concerns how cells survive the deleterious effects of enhanced mutagenesis. One proposed strategy is the differentiation of a subpopulation of transiently hypermutable cells. This study investigates a previously discovered hypermutable cell subpopulation (HMS) postulated either to underlie most stress-induced mutagenesis in *E. coli* or only a small fraction of it. First, improved methods allow estimation of mutations per genome accumulated during HMS-generated bursts of mutagenesis and show numbers compatible with fitness after the HMS state. Second, two lines of evidence presented support models in which the HMS is central to this stress-induced mutagenesis pathway. Third, a specific model, with general consequences, for HMS differentiation is discussed.

## Introduction

Stress-induced mutational processes are responses to growth-limiting environments whereby mutations are produced at an accelerated rate, some of which may confer a growth advantage. The study of stress-induced-mutagenesis mechanisms is expanding our understanding of genome instability and cellular and organismal adaptability to environmental challenges (reviewed [1],^2^). Whereas classical spontaneous mutations occur in proliferating cells, in a generation-dependent manner, and before cells encounter an environment in which the mutations might prove useful [e.g.],[3],[4,5], stress-induced mutations occur in growth-limiting environments, often under the control of stress responses, *via* pathways different from those observed in rapidly proliferating cells (reviewed [2]). Stress-induced mutagenesis may potentially accelerate evolution specifically when cells/organisms are maladapted to their environments, i.e., when they are stressed. Stress-induced mutagenesis mechanisms appear to be widespread and important in nature. The vast majority of 787 natural isolates of *E. coli* show induction of mutagenesis by starvation stress [6]. Stress-induced mutagenesis mechanisms present appealing models for mutagenesis underlying evolution of antibiotic resistance, evasion of the immune response by pathogens, aging, and for genomic instability underlying tumor progression and resistance to chemotherapeutic drugs, all of which are fueled by mutations and occur in stress-provoking environments (reviewed by [2],[7]).

There are multiple molecular mechanisms of stress-induced mutagenesis, observed in different organisms, strains and stresses, but many share important common elements, including control by cellular stress responses (reviewed [2]). In the *Escherichia coli* Lac assay [8], the mechanism of mutagenesis is a stress-response-controlled switch from high-fidelity to error-prone DNA double-strand-break repair during stress, described below. In the Lac assay, cells carrying a chromosomal deletion of the *lac* genes and a *lac* +1 frameshift allele in an F' episome are starved for carbon on solid minimal lactose medium. Over time, Lac^+^ mutant colonies appear. Many of those visible at day two carry generation-dependent spontaneous mutations that occurred during growth of the culture prior to plating (the Lac^+^ mutants take two days to form a colony on the selective medium) [8]. Colonies that appear on later days are stress-induced mutants, which form after exposure to starvation-induced stress [8],[9] in a process requiring the RpoS general- or starvation-stress response [10],[11].

At least two independent mechanisms produce the stress-induced Lac^+^ colonies: Lac^+^ “point mutagenesis” and stress-induced gene amplification. Point mutagenesis dominates during the first week of incubation and creates compensatory -1 frameshift mutations [12],[13]. Tandem amplification of the *lac* region to 20–100 copies represents ∼40% of Lac^+^ colonies at day eight of incubation, and increases thereafter [14],[15]. Amplification allows growth on lactose because multiple copies of the weakly functional mutant *lac* gene produce enough beta-galactosidase activity to restore growth. Both processes are stress-induced and require the RpoS-controlled general-, stationary-phase- or starvation-stress response [11]. This paper focuses on the mechanism of stress-induced point mutagenesis. Readers are referred to [16]–[18] for recent reviews of the mechanism(s) of stress-induced amplification, and its relevance to genome instability in cancer as well as copy-number variations ubiquitous in human and other genomes.

Work from our lab has provided support for a model in which stress-induced point mutagenesis results from DNA polymerase errors made during acts of DNA double-strand-break repair (DSBR), which is switched to a mutagenic mode, using an error-prone DNA polymerase, specifically during stress [19]:

First, point mutagenesis requires homologous-recombinational (HR)-DSBR proteins RecA (EG10823), RecBC (EG10824 and EG10825), RuvA (EG10923), RuvB (EG10924), and RuvC (EG10925) [20]–[22], and it and stress-induced amplification are greatly stimulated by DSBs made using a regulatable I-SceI (P03882) endonuclease in vivo [19]. Induction of I-SceI cuts next to *lac* increases mutation rate over 1000-fold; whereas I-SceI-induced DSBs made in another molecule provoke *lac* reversion only 3-fold. However the DSBs made in a different molecule from *lac* can again stimulate *lac* reversion dramatically if one end of the broken DNA molecule contains DNA identical to DNA next to *lac*, such that homologous repair with the *lac* region can be initiated [19].

Second, I-SceI-stimulated stress-induced Lac^+^ point mutagenesis occurs by the same mechanism as “normal” stress-induced mutagenesis in that both require the HR-DSBR proteins [19], the RpoS general-stress-response transcriptional activator [10],[11],[19], induction of the SOS DNA-damage response [19],[23], and functional *dinB* (EG13141), encoding DNA polymerase (Pol) IV [19],[24] of the Y-superfamily of trans-lesion, error-prone DNA polymerases [25]. These specialized DNA polymerases insert bases opposite otherwise replication-blocking lesions in DNA with reasonably good fidelity, but have low fidelity and are error-prone when synthesizing on undamaged template DNA. Both the SOS response [26],[27] and the RpoS response [10] upregulate *dinB*, 10- and about 2-fold, respectively. *dinB* upregulation might account for some or all of the requirement for induction of the SOS and RpoS responses for stress-induced point mutagenesis, though this has not been demonstrated.

The similarity of the proteins required for I-SceI-stimulated and “spontaneous” stress-induced mutagenesis argues that both occur by the same mechanism, as does the finding that I-SceI-induced and “normal” stress-induced Lac^+^ point mutations are indistinguishable in their Lac^+^ mutation sequences [19]. All of these data support the idea that stress-induced mutagenesis occurs *via* error-prone HR-DSBR in which DinB/Pol IV has been licensed to participate in the HR-DSBR reaction [19].

Finally, HR-DSBR is not always mutagenic but rather switches to a mutagenic mode, with DinB/Pol IV participating, under stress. This switch is controlled either by entry of cells into the stationary phase, or, in log-phase cells if the RpoS stationary-phase stress-response transcriptional activator is expressed inappropriately [19]. In both cases, the SOS response should often already be induced by the DSB, given that even well repaired DSBs induce SOS efficiently [28]. (Alternative models for stress-induced Lac point mutagenesis are discussed below.)

Thus, mutagenesis is limited to times of stress *via* its coupling to two stress responses (SOS and RpoS). Mutagenesis is potentially also restricted in genomic space *via* being coupled to potentially localized DNA synthesis during DSBR [19]. Both of these restrictions may protect populations from deleterious effects of mutagenesis, and both themes are evident in many different mutagenesis mechanisms in organisms from phage to human, and so appear to be general mutational/evolutionary strategies [reviewed, 2, and Discussion].

In this paper, we investigate a third level of restriction/limitation or regulation of mutagenesis: its limitation to a subpopulation of stressed cells while the main population appears to be unaltered. In the Lac system, there is strong evidence that a subpopulation of cells becomes transiently hypermutable, resulting in mutations in genes throughout the genome. First, *E. coli* [29]–[31] and Salmonella [32] Lac^+^ stress-induced point mutants show, respectively, ∼20 and ∼50 times more loss-of-function mutations in chromosomal genes throughout their genomes than are found in Lac^−^ cells that starved for the same length of time: their Lac^−^ neighbors from the same Petri plates. Those Lac^−^ cells represent the main population whereas some or all of the Lac^+^ mutants arose from a more mutable subpopulation: a hypermutable cell subpopulation (HMS). The evidence that the hypermutability of this HMS is transient is, second, that once the cells have become Lac^+^, they do not have elevated spontaneous [29]–[31] or stress-induced [33] mutation rates. Moreover, when whole colonies of the initial stress-induced Lac^+^ mutants were picked and analyzed these colonies were mostly pure, not mosaic, for the unselected mutations that they carried, indicating that they accrued the unselected chromosomal mutations during or before acquiring the Lac^+^ mutation, not after, further showing that the mutability was transient [29]. The possible evolutionary significance of differentiation of a HMS is that this may protect most members of a clone from the deleterious effects of inducing mutagenesis, an advantage should nutrients suddenly become available, while simultaneously allowing the exploration of evolutionary space when maladapted to an environment.

Although there is consensus in the field regarding the existence of the HMS, both the extent of HMS-cell mutagenicity and the importance of the HMS to most stress-induced mutagenesis are currently unresolved. First, the HMS could either be important or not. On the one hand, the HMS has been hypothesized to give rise to essentially all stress-induced Lac^+^ point mutants [29], whereas on the other hand, other models suggest that the HMS may contribute to only a small minority, ∼10% or so, of Lac^+^ point mutants [30],[32], and so be relatively unimportant. Second, it has been argued that too much mutagenesis would occur in the HMS state for it to be adaptive [34]. Here, we first estimate the number of mutations per genome in *E. coli* cells derived from the HMS and find a level that need not preclude fitness. Second, we provide two lines of experimental support and mathematical modeling that support the idea that the HMS generates most or all, not just a minority of, Lac^+^ stress-induced point mutants. Finally, we consider a model for a mechanism by which the HMS is differentiated.

## Results

### Numbers of Unselected Secondary Mutations per Genome

To better understand the potential fitness impact of cells' entering into a transient hypermutable state, we wished to estimate the number of mutations expected per genome in cells that have undergone stress-induced mutagenesis. Numbers of unselected secondary mutations among Lac^+^ mutants are reported in previous studies, but were not used previously to estimate the numbers of mutations per genome. We used the previous data to estimate numbers of mutations per genome (Table 1 and Text S1), and we found that the answer differs between studies that used different organisms and methods for assaying unselected secondary mutations among the Lac^+^ stress-induced mutants. Whereas the data from three studies in *E. coli* [29],[30],[35] can be extrapolated to imply that about one unselected mutation cluster (of one or more mutations, discussed below) occurs per genome, in addition to the Lac^+^ mutation (Table 1/Text S1,), the data from a study using *Salmonella enterica* and a different mutation-assay method can be extrapolated to indicate about 2.5 unselected mutation clusters, in addition to Lac^+^, per genome (Table 1/Text S1). In the previous *E. coli* studies, the secondary mutations were detected by direct transfer of Lac^+^ colonies (either by replica-plating or patching) directly from the lactose-selection plates to specific indicator media that, for example, showed a different color colony for fermentation-defective mutants. This technique is likely to miss some mutants that are overlapped with wild-type colonies. By contrast, in the previous Salmonella study [32], the authors screened for auxotrophic mutations, using a more sensitive technique. They picked the Lac^+^ colonies and purified them by streaking, patched them into grids, grew, then replica-plated to media that would indicate auxotrophic mutations by failure of the patch to grow on medium lacking amino acids and bases. This technique is likely to produce fewer false negatives due to overlap of mutant with non-mutant colonies. To understand whether their somewhat different result arose from use of a different organism or the different mutation-detection method, we used their presumably more sensitive method with *E. coli* to improve estimates of unselected secondary mutations per stress-induced mutant genome.

**Table 1:** Estimates of Mutation Clusters per Genome of Lac^+^ Stress-Induced Mutants^a^.

Data Source	Method^b^	Organism	Screen^c^	Approx. basepairs targeted^d^	SecondaryMutants/Lac^+^ Mutant	Secondary Mutant Frequency	Extrapolated Mutation Clusters/Genome^e^
[29]	DT	*E. coli*	Mal^−^	3178	31/42,617	7.3×10^−4^	1.1
[30]	DT	*E. coli*	Mal^−^	3178	2/3168	6.3×10^−4^	0.92
[35]	DT	*E. coli*	Mal^−^	3178	10/15,009	6.7×10^−4^	0.97
This study^f^	PP	*E. coli*	Mal^−^	3178	8/3437	2.3×10^−3^	3.4
[29]	DT	*E. coli*	Xyl^−^	1811	22/42,617	5.2×10^−4^	1.3
[35]	DT	*E. coli*	Xyl^−^	1811	12/15,009	8.0×10^−4^	2.0
This study^f^	PP	*E. coli*	Xyl^−^	1811	3/3437	8.7×10^−4^	2.2
[32]	PP	*S.enterica*	Aux	33,000	16/926	1.7×10^−2^	2.5
This study^f^	PP	*E. coli*	Aux	28,920	28/3437	8.1×10^−3^	1.3

a In all of the studies cited, the frequency of one or more classes of chromosomal unselected secondary mutations were ascertained among Lac^+^ stress-induced mutants, the number of base-pairs that could be mutated to produce the mutant phenotype assayed was estimated (Text S1), and the number of mutations expected per all of the basepairs in the genome was then extrapolated. These estimates are based on the assumption that all Lac^+^ stress-induced mutants had an equal probability of accumulating secondary mutations, i.e., that a single mutable population produces stress-induced mutants. Other models and their consequences are discussed in the Discussion.

b Direct transfer (DT) and purify-and-patch (PP) methods for identifying secondary mutants among Lac^+^ mutants are described in the text.

c Phenotype assayed for when screening for secondary mutants. Mal^−^, unable to ferment maltose; Xyl^−^, unable to ferment xylose; Aux, auxotrophic mutants.

d The approximate numbers of basepairs that when mutated can lead to the phenotypes screened are estimated in Text S1, except for Salmonella auxotrophs, which we estimate by comparison with *E. coli* to involve 84 genes of a total size of about 99,000bp, one third of which, or 33,000bp, would be predicted to give a phenotype when mutated (see Text S1).

e The mutations observed per basepair targeted are extrapolated to the 4,639,221 bp *E. coli* genome. For *S. enterica* we took a genome size of 4,857,432 [82]. These figures represent the number of predicted mutation clusters (of one or more mutations) in addition to the Lac^+^ mutation in these cells.

f These are the combined data from two strains. Each strain served as a negative control, in which there was no cleavage of DNA with the endonuclease I-SceI, for experiments in which the frequency of secondary mutations was assayed in cells that express I-SceI and carry an I-SceI cutsite, and which we show experience DNA cleavage. The two negative-control strains, SMR6276 and SMR6277, either express the enzyme but have no cutsite (“Enzyme only” strain) or have neither the cutsite nor the I-*Sce*I gene under the control of the chromosomally engineered P*_BAD_* promoter (“P*_BAD_* only” strain), and the data from each strain separately are shown in Table 3.

First, we show that for *E. coli*, the purify-and-patch method is more sensitive than direct transfer by replica plating for three mutant phenotypes scored (Table 2). Second, using the purify-and-patch method for all of the results presented here, we observed 8/3437 (2.3×10^−3^) Mal^−^ mutations per Lac^+^ cell (Table 1). If these occurred in 3178bp (Text S1), then we estimate 3.4 mutations or mutation clusters in addition to Lac^+^ per 4,639,221bp *E. coli* genome (Table 1). Third, we found 3/3437 (8.7×10^−4^) Xyl^−^ mutants per Lac^+^ point-mutant colony, implying 2.2 mutation clusters in addition to Lac^+^ per genome (Table 1). Fourth, we assayed for auxotrophic mutations targeting 72 loci providing a mutation target of 28,920bp (Text S1). We observed 28/3437 (8.1×10^−3^) auxotrophic mutants per Lac^+^ point mutant (Table 1). This extrapolates to 1.3 mutations or mutation clusters in addition to that conferring Lac^+^ per *E. coli* genome (Table 1). These estimates per genome assume that all Lac^+^ stress-induced point mutants are equally likely to acquire secondary mutations. If only some do then the number of mutation clusters per genome would be higher in those that do (Discussion).

**Table 2:** Sensitivity of the Purify-and-Patch Method.

Mutation-Detection Method (source of data)	Mutation phenotype	Secondary mutant frequency among Lac^+^ mutants	
Direct transfer by replica plating^a^ ([29])	Mal^−^	31/42,617 =	7.3×10^−4^
	Xyl^−^	22/42,617 =	5.2×10^−4^
Direct transfer by replica plating^b^ (this study)	Auxotrophic	Could not detect among 10,687^c^	
Purify-and-patch^b^ (this study)	Mal^−^	8/3437 =	2.3×10^−3^
	Xyl^−^	4/3437 =	1.2×10^−3^
	Auxotrophic	28/3437 =	8.1×10^−3^

a Strain FC40.

b Strain SMR6277. This strain and FC40 are shown not to have different frequencies of secondary mutations when assayed by the same method (Table S1).

c This result probably does not mean that the frequency of auxotrophs was less than 9×10^−5^ (1/10,687) but rather that the method of direct transfer via replica plating is particularly ill suited to detection of phenotypes that result in the inability to form a colony.

The somewhat higher estimates of secondary mutation clusters per genome in this study compared with those estimated from previous *E. coli* data (Table 1) is expected to reflect the more sensitive “purify-and-patch” method used here, but alternatively, might reflect the fact that the strains used here differ slightly from that used previously. Unlike the previously used strain, the present strains carry either the chromosomal P*_BAD_*I-*Sce*I-expression cassette (“Enzyme-only” strain) or the P*_BAD_* promoter replacing the phage lambda attachment site (*att*λ) in the chromosome (“P*_BAD_*-only” strain). These strains are negative-control strains for experiments presented below. In Table S1, we show that these slight strain differences are not the relevant variable. We assayed the P*_BAD_*-only strain for loss-of-function mutations among Lac^+^ revertants by direct transfer via replica plating straight from lactose plates onto indicator and selective plates as performed in [29]. We find no significant difference in the proportion of Lac^+^ mutants with secondary mutations from those previously reported, *p* = 0.697, (z-test with Yates correction) (Table S1). This rules out the unlikely possibility that the new strains used in this study might have shown enhanced secondary mutation for some reason specific to their genotype, and so confirms that the different mutation-assay method used here is responsible for the somewhat higher frequency of secondary mutations observed relative to previous *E. coli* studies [29],[30],[35].

Taken together, the data indicate between about one and 3.4 mutation clusters in addition to Lac^+^ per stress-induced-mutant cell genome.

### Clustering of Mutations

Mutations in the Lac system appear to be clustered locally in the DNA [35] such that the estimates above are likely to pertain to numbers of mutation clusters per genome. We can make a rough estimate of the number of mutations per mutation cluster from data on the apparent clustering of Lac^+^ mutants with the linked mutations in the *codAB* genes (EG11326 and EG11327) 10kb from *lac*. Previously, loss-of-function mutations in the *codAB* genes, which confer resistance to the nucleotide analogue 5-fluorocytosine (5-FC^R^) were shown not to form independently of Lac^+^ mutations, whereas unlinked chromosomal mutations did, in a study using the direct-transfer-by-replica-plating method [35]. (Note that two *E. coli* loci confer 5-FC^R^ when mutated, but only *codAB* mutations confer 5-FC^R^ without also conferring resistance to 5-fluorouracil [5-FU], which is how these mutations were distinguished [35]). Here, we re-quantify coincident mutation of *codAB* and *lac* using the purify-and-patch method for detecting 5-FC^R^ mutants (Table 3). We observe that 5-FC^R^ (5-FU-sensitive) mutations in *codAB* are more frequent among Lac^+^ mutants than are unlinked mutations (Table 3, first two columns). These and the previous data [35] imply that *codAB* mutations cluster with *lac* mutations. In the Text S1, we estimate cluster size, and then estimate mutations per cluster from the data in Table 3, as about 1.67 mutations per cluster.

**Table 3:** Effect of I-SceI Endonuclease on Coincidence of Secondary Mutations with Lac^+^ Point Mutations.

Replicon carrying the unselected mutation	Mutant Phenotype	Secondary Mutant Frequency Among Lac^+^ Mutants^a^		
		Lac^+^ No-DSB strain (P*_BAD_* Only^b^)	Lac^+^ No-DSB strain (Enzyme Only^c^)	Lac^+^ DSB strain (Enzyme and Cutsite^d^)
F'	5-FC^R^	14/1693 (8.3×10^−3^)	12/1744 (6.9×10^−3^)	41/2604 (1.6×10^−2^)
Chromosome	Mal^−^	4/1693	4/1744	16/2604
	Xyl^−^	1/1693	2/1744	15/2604
	Auxotroph	14/1693	14/1744	63/2604
	Mucoid^e^	1/1693	3/1744	10/2604
	Total chromosomal	20/1693 (1.2×10^−2^)	23/1744 (1.3×10^−2^)	104/2604 (4.0×10^−2^)

a In this and all of the tables and figures in this paper, stress-induced Lac^+^ colonies were divided into point mutants (compensatory frameshift revertants) and *lac*-amplified clones per [14], and only the point mutants were screened for secondary mutations. Because stress-induced *lac*-amplifications are not associated with secondary mutations (or a HMS) [14], this controls for differential effects of any of the treatments studied on point mutagenesis and amplification.

b Strain SMR6277. This strain is a negative control that expresses neither I-SceI endonuclease nor carries the I-SceI cutsite, and so does not make I-SceI-mediated DNA double-strand breaks (DSBs). It is a negative control for the “Enzyme and Cutsite” strain SMR6280 which expresses I-SceI from a chromosomal regulatable promoter P*_BAD_* replacing the phage lambda attachment site (Δ*att*λ::P*_BAD_*I*-Sce*I) and carries an I-SceI site, and makes DSBs. This strain has the P*_BAD_* promoter insertion without the I-*Sce*I gene, Δ*att*λ::P*_BAD_*, and so is designated “P*_BAD_* only”).

c Strain SMR6276. This strain is a second negative control for the I-SceI-mediated DSB-producing strain SMR6280. This “Enzyme-only” strain carries the Δ*att*λ::P*_BAD_*I*-Sce*I expression cassette but no I-SceI cutsite.

d Strain SMR6280, with both the chromosomal Δ*att*λ::P*_BAD_*I*-Sce*I expression cassette and the F'-located I-SceI cutsite, makes I-SceI-induced DSBs near *lac* [19]. This strain shows greatly increased Lac^+^ stress-induced mutagenesis ([19] and shown here, Figure 3).

e “Mucoid” colonies had a mucoid appearance on minimal M9 glucose plates and did not form colonies on either maltose or xylose MacConkey medium.

### Lac^+^ Mutations from HMS Cells Are Like Most Stress-Induced Lac^+^ Mutations

**Figure 1:**
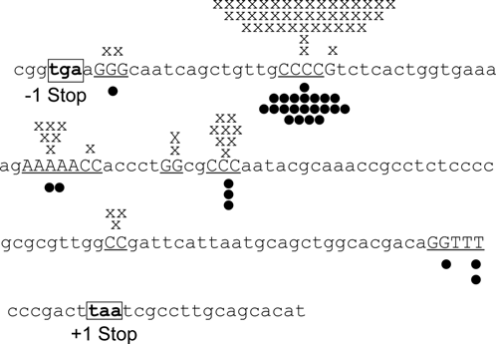
Lac^+^ Mutation Sequences in HMS-Descended Cells. The sequences of stress-induced Lac^+^ frameshift-reversion mutations are nearly all -1 deletions in small mononucleotide repeats at the positions shown. Those from cells carrying chromosomal “secondary” mutations, detected in our screens, (•, this study) are indistinguishable from stress-induced Lac^+^ frameshift reversions from cells without detected secondary mutations (X, data from [12],[13]). The 30 new mutants sequenced (•) were identified in a previous screen for Lac^+^ mutants with chromosomal loss-of-function mutations [29] conferring the following phenotypes: Mal^−^ (15 mutants); Xyl^−^ (10 mutants); minimal temperature sensitive (TS), which grow on minimal medium at 37° but not at 42° (1 mutant); Mal^−^ Xyl^−^ double mutants (3 mutants); and Mal^−^ minimal TS (1 mutant).

In models in which the HMS is predicted to produce only a 10% minority of the Lac^+^ stress-induced mutants, the mutations that occur in the HMS and give rise to Lac^+^ phenotype are proposed to occur via a different molecular mechanism from that that generates the 90% majority of stress-induced Lac^+^ mutations [30],[32]. If true, those Lac^+^ mutations that arise from HMS cells might be predicted to display different reversion-mutation sequences from the majority of stress-induced Lac^+^ mutations. We examined the Lac^+^ mutation sequences from stress-induced mutants that demonstrably descended from the HMS, as seen by their carrying an unselected “secondary” chromosomal mutation, and compared these with the published sequences of stress-induced Lac^+^ mutations [12],[13]. We sequenced a 250bp region spanning the +1 frameshift mutation of the *lacI*-*lacZ* (EG10525 and EG10527) fusion gene from 30 independent Lac^+^ point-mutant isolates carrying secondary mutations. We find that the mutation sequence profile is indistinguishable from those previously reported for stress-induced mutants [12],[13]: dominated by -1 deletions in small mononucleotide repeats with a hotspot at the position of the initial *lac* frameshift allele (Figure 1). These data support the hypothesis that the mechanism of mutagenesis in the HMS cells is similar to or the same as the stress-induced mutagenesis mechanism that generates all or most Lac^+^ point mutations. This distinctive mutation spectrum differs from spontaneous generation-dependent reversions of this *lac* allele, which are more heterogeneous [12],[13]. Summarized in Table S2, these include about half -1 deletions at mononucleotide repeats, and half carrying -1's not at repeats, 2–8 bp insertions, and large insertions and deletions. Instead, the stress-induced Lac^+^ frameshift-reversion sequences resemble the frameshift component of the error spectrum of DinB/Pol IV [36],[37] which is responsible for ≥85% of Lac^+^ point mutations in this assay system [24].

### Genome-Wide Mutagenesis Is Inseparable from Stress-Induced Lac^+^ Mutagenesis upon DNA Cleavage in Vivo

The previous demonstration that stress-induced mutations in the Lac system result from error-prone DNA double-strand-break repair (DSBR) and are greatly stimulated by creation of DSBs next to *lac* in vivo [19], allowed us to make a second test of whether the HMS underlies most stress-induced mutagenesis. In that study [19], DSBs generated near the *lac* gene by the endonuclease I-SceI were shown to increase Lac^+^ mutant frequency dramatically: more than 1000-fold above the levels seen in *traI* (P14565) endonuclease-defective mutants that cannot make nicks in the transfer origin of on the F', and more than 50-fold above levels in TraI^+^ cells (TraI-generated nicks usually promote mutations in this assay but are more than compensated for by I-SceI-generated DSBs [19]). Most importantly, the I-SceI-induced mutations occurred via the main mechanism of mutagenesis that operates normally (without I-SceI-induced DSBs), not a minority mechanism as shown by the following: the Lac^+^ sequences were the same; and the mechanism of mutagenesis with I-SceI induction specifically required RecA, RecB and Ruv DSB-repair proteins; DinB error-prone DNA polymerase; the RpoS transcriptional activator of the general stress response; and a functional SOS DNA-damage response, all of which are specifically required for the main mechanism of stress-induced mutagenesis in wild-type cells [19]. Therefore, stimulation of stress-induced mutagenesis by I-SceI cleavage increases the activity of the predominant, normal stress-induced-mutagenesis mechanism. We exploited this fact to examine whether this major increase in Lac^+^ mutagenesis by I-SceI cleavage of DNA near *lac* happens independently of the HMS, or inseparably from the HMS, by measuring the frequencies of chromosomal mutations among I-SceI-induced Lac^+^ mutants.

**Figure 2:**
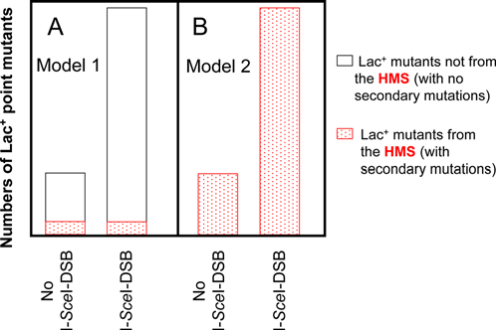
Different Models for the Role of the HMS in Mutagenesis: Predictions for How Mutagenesis Is Enhanced by I-SceI Endonuclease. (A) Model 1: the HMS generates few stress-induced Lac^+^ mutants and does so via mechanism(s) not relevant to most stress-induced mutagenesis. These models predict that when the main DSB-repair-dependent mechanism of stress-induced mutagenesis (open bars) is stimulated by I-SceI-mediated DSBs made near *lac* in vivo [19], Lac^+^ mutagenesis will increase from cells not undergoing genome-wide mutagenesis (open bars). This would cause a decrease in the frequency of genome-wide secondary mutations (present only in the red-dotted fraction) per total Lac^+^ mutant (open and red-dotted total). (B) Model 2: the HMS generates most/all stress-induced Lac^+^ mutants. Models in which genome-wide mutagenesis necessarily accompanies most/all stress-induced Lac reversion predict that the proportion of Lac^+^ mutants with additional chromosomal mutations (red dotted) will not decrease when mutation is stimulated by I-SceI-induced DSBs.

The idea is as follows: if only 10% of Lac^+^ mutagenesis were associated with secondary mutagenesis of unselected genes throughout the genome (proposed [30],[32]), and if I-SceI increased the efficiency of most stress-mutagenesis (proposed to form HMS-independently [30],[32]), then I-SceI-induction of stress-induced Lac^+^ mutagenesis would be expected to increase Lac^+^ mutagenesis without also increasing secondary mutagenesis of unselected genes throughout the genome (illustrated in Figure 2A, Model 1). I-SceI should “uncouple” Lac^+^ mutagenesis from secondary mutations such that the frequency of secondary mutations per Lac^+^ mutant should decrease (Figure 2A). On the other hand, if all stress-induced Lac^+^ mutagenesis occured in HMS cells [29],[35],[38], then the frequency of secondary mutations per Lac^+^ mutant cell should be unchanged (Figure 2B, Model 2). I-SceI cleavage might increase the size of the HMS (Discussion), but would not decrease its mutagenicity.

**Figure 3:**
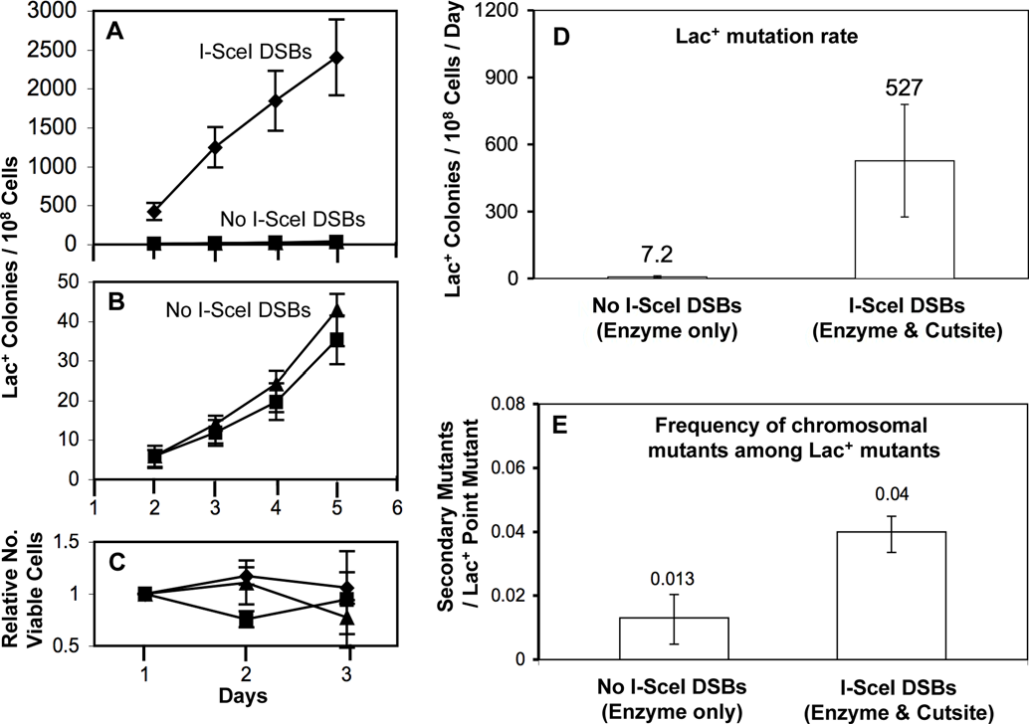
Lac^+^ Mutations and Genome-Wide Mutagenesis Remain Coupled during I-SceI-Mediated Stimulation of Stress-Induced Mutagenesis. (A) I-SceI-mediated DNA cleavage near the *lac* gene stimulates stress-induced Lac reversion. Representative experiment. Strains: SMR6280; I-SceI DSBs (enzyme+cutsite) (♦), SMR6276; No I-SceI DSBs (enzyme only) (▪), SMR6281; No I-SceI DSBs (cutsite only) (▴). (B) Data from (A) displayed with the *y* axis expanded. (C) Viable cell measurements of the Lac^−^ cells during the experiment shown in A and B show no significant growth or death of the strains during the experiment. Because it takes two days for a Lac^+^ cell to form a colony on lactose minimal medium, these viable cell measurements on days 1, 2 and 3 pertain to Lac^+^ colonies visible on days 3, 4 and 5, respectively. (D) Stress-induced mutation rates are increased by I-SceI action near *lac*. Data from two independent experiments, mean±range (error bars). Lac^+^ mutations accumulated over five days of selection in a strain without I-SceI-induced DSBs (No I-SceI DSBs, SMR6276), and in an I-SceI-mediated-DSB-inducible strain (I-SceI DSBs, SMR6280), showing a ∼70-fold increase in mutation rate when both I-SceI enzyme and its cutsite near *lac* are present. (E) Frequencies of secondary chromosomal mutations (auxotrophic mutants plus Mal^−^, Xyl^−^, and mucoid from Table 3) per Lac^+^ point mutant are not decreased by I-SceI-mediated DSB stimulation of mutagenesis. The slight increase in the frequency of secondary mutations in the I-SceI-cut-induced strain (I-SceI DSBs, SMR6280) relative to the non- I-SceI-cut-inducible strain (No I-SceI DSBs, SMR6276) is significant: *p* = 0.001 (z-test with Yates correction). Error bars show 95% confidence limits for binomial populations.

As seen previously [19], we found that a strain carrying both a regulatable chromosomal expression cassette of the I-SceI enzyme and its cutsite on the F' plasmid near *lac* showed a 70-fold increase in Lac^+^ mutation rate (Figure 3A,D) above that promoted by TraI-dependent DNA breaks at the transfer origin of the F' in the “wild-type” control cell. As previously, this was not seen in controls with only the enzyme expressed (no cutsite) or only the cutsite present (no enzyme) (Figure 3A,B,D). Lac^+^ point-mutant colonies from days four and five were assayed for unselected loss-of-function secondary mutations (Materials and Methods, and above).

First, we found that chromosomal loss-of-function mutations conferring inability to ferment maltose (Mal^−^), or xylose (Xyl^−^), or a mucoid-colony or auxotrophic phenotypes were not decreased among I-SceI-induced Lac^+^ point mutants as compared with negative-control strains that did not experience cleavage by I-SceI: the “enzyme-only” or “P*_BAD_*-only” controls (Figure 3E and Table 3). Thus, genome-wide mutagenesis was not uncoupled from Lac^+^ point mutagenesis (Figure 3E and Table 3) even though there was a 70-fold increase in mutagenesis caused by cleavage of DNA near *lac* by I-SceI (Figure 3A–D). This indicates that the main mechanism of Lac^+^ point mutagenesis does not occur independently of the HMS. This supports the hypothesis that Lac^+^ point mutagenesis is inseparable from the HMS (Model 2 of Figure 2B).

Second, there is a small but statistically significant increase in chromosomal secondary mutation frequencies among Lac^+^ point mutants accompanying I-SceI-mediated DNA breakage. This is discussed below (Discussion).

### Expression of I-*Sce*I Affects Mutation Only with a Cutsite Present

We assessed the possibility that the induction of I-SceI enzyme might be mutagenic in its own right and therefore might affect the proportion of chromosomal mutations independently of the formation of a DSB. We tested isogenic strains that lack the I-SceI cutsite, and either carry the chromosomal I-SceI-expression cassette Δ*att*λ::P*_BAD_*I-*Sce*I (“Enzyme only”) or carry the chromosomal regulatable promoter without the I-*Sce*I gene, Δ*att*λ::P*_BAD_* (“P*_BAD_* only”), for secondary chromosomal mutations. The proportion of Lac^+^ point mutants carrying a chromosomal secondary mutation was no different for cells expressing I-*Sce*I with no cutsite (enzyme only) compared with the P*_BAD_*-only strain, *p* = 0.697, (z-test with Yates correction) (Table 3). This demonstrates that I-*Sce*I expression does not affect frequencies of chromosomal mutations unless an I-SceI cutsite is also present.

### I-SceI-Induced DSBs Do Not Convert All Cells into HMS Cells

Previous work from our lab showed that cleavage of DNA near *lac* by I-SceI and repair of the break were not sufficient to increase stress-induced Lac reversion; in addition, the cells had to be either in stationary phase, or expressing the stationary-phase- (general- or starvation-) stress-response transcriptional activator protein RpoS (EG10510) (σ^S^, a sigma factor for RNA polymerase) [19]. Thus, repair of DSBs is not always mutagenic, but becomes so when cells activate their RpoS stress response. As expected from this result, and from the finding that Lac^+^ and genome-wide secondary mutations are coupled (Table 3, Figure 3E), we found that Lac^−^ unstressed cells do not show dramatically increased secondary mutation frequencies upon I-SceI induction (Table 4). Our results showing no secondary mutations among the 4000 Lac^−^ unstressed cells assayed (Table 4) cannot distinguish whether secondary mutations were increased at all by I-SceI in unstressed cells, but do reveal that secondary mutations are not increased to levels seen among Lac^+^ mutants. That is, as expected, cleavage near *lac* with I-SceI is not sufficient to convert unstressed cells into HMS cells.

**Table 4:** Secondary Mutations Associated with Different Populations with DSBs.

		Secondary Mutant Frequency Among Lac^+^ Mutants DSB-Inducible Strain (Enzyme and Cutsite^a^)		
Replicon carrying the unselected mutation	Mutant Phenotype	Lac^−^ Unstressed^b^	Lac^−^ Stressed^c^	Stress-Induced Lac^+^ Point Mutants^d^
F'	5-FC^R^	0/4000	0/4000	41/2604 (1.6×10^−2^)
Chromosome	Mal^−^	0/4000	0/4000	16/2604
	Xyl^−^	0/4000	0/4000	15/2604
	Auxotroph	0/4000	0/4000	63/2604
	Mucoid	0/4000	0/4000	10/2604
	Total chromosomal	0/4000 (<2.5×10^−4^)	0/4000 (<2.5×10^−4^)	104/2604 (4.0×10^−2^)

a Strain SMR6280

b Cells not starved but grown into colonies and assayed by the purify-and-patch method (Materials and Methods).

c Cells that starved on lactose plates but did not become Lac^+^ (recovered per [29], discussed in text) assayed by the purify-and-patch method (Materials and Methods).

d Data from Table 3.

Second, perhaps surprisingly, we also found that not all Lac^−^ stressed cells are converted into HMS cells upon I-SceI induction. Lac^−^ stressed cells were recovered from the lactose selection plates by sampling agar from between visible Lac^+^ colonies at day three of incubation, re-suspended and plated on non-selective LBH rifampicin X-gal glucose medium. (Day-three starving cells correspond to day-five Lac^+^ colonies because colony formation on the lactose medium takes two days after acquisition of the Lac^+^ mutation [8],[9].) The colonies were then assayed for loss-of-function mutations conferring 5-FC^R^, Mal^−^, Xyl^−^, mucoid and auxotrophic phenotypes. Our results showing no secondary mutations among the 4000 Lac^−^ stressed cells assayed (Table 4) show that secondary mutations are not increased to levels seen among Lac^+^ mutants. That is, even in starving cells, cleavage near *lac* with I-SceI apparently does not convert every cell into a HMS cell within the time-frame of an experiment.

Thus the elevated mutability observed among the DSB-induced Lac^+^ mutants is specific to a subpopulation of cells (i.e., an HMS) and induction of I-SceI-DSBs is not sufficient to render the whole population hypermutable.

## Discussion

### Importance of the HMS

The results presented here provide evidence supporting the hypothesis that a previously detected HMS [29]–[32] is important to the genesis of most stress-induced Lac^+^ revertants, not merely a small fraction as had been suggested [30],[32]. First, the unique sequence spectrum of the majority of stress-induced Lac^+^ reversion mutations was also observed in those Lac^+^ mutants demonstrably descended from the HMS: those carrying phenotypically-detectable secondary mutations in their genomes (Figure 1), implying that HMS-descended and most stress-induced Lac^+^ reversions form via the same mechanism. Second, the main mechanism of stress-induced mutagenesis in the Lac system is an RpoS-controlled switch to error-prone DSBR causing mutations at the sites of repair [19], and requiring HR-DSBR proteins, RpoS, the SOS response, and DinB low-fidelity DNA polymerase ([19] and reviewed [2]). Stimulation of stress-induced HR-DSBR-associated Lac reversion by DSBs delivered next to *lac* in vivo did not decrease the frequency of secondary mutants among the Lac^+^ mutants (Table 3, Figure 3) indicating that this main mechanism was inseparable from the HMS (per Figure 2B).

Mathematical modeling of previous data led two groups to favor the hypothesis that the HMS produced only 10% of stress-induced Lac^+^ revertants in *E. coli* [30], and in a similar but not identical experimental system in *Salmonella enterica* [32]. The other 90% of Lac revertants were suggested to arise independently of, and by some other mutagenic mechanism(s) than operates in, the HMS. In a prominent alternative model, the main 90% were proposed to form in cells with no increase in mutation rate relative to that in non-stressed cells, by “standard” generation-dependent mutational processes. The HMS was proposed to generate only few Lac^+^ mutants via co-amplification of *dinB* (EG13141), encoding error-prone DNA pol IV, with *lac* causing a mutator state [32].

The hypothesis that only 10% of Lac^+^ mutants arose from the HMS (whether via *dinB* amplification [32] or otherwise [30]) was based on estimation of mutability in Lac^+^ mutants with no phenotypically detected chromosomal secondary mutations (Lac^+^ “single” mutants) and finding a lower estimated value than in similar estimates from “double” mutants (Lac^+^ revertants with one phenotypically detected secondary mutation) [29],[30]. This was interpreted in terms of the HMS generating most double, triple and multiple mutants but few (only 10%) of the Lac^+^ single mutants [30], an interpretation not supported by the data presented here. We believe that the previous modeling [30] did not allow for cells to exit the HMS immediately upon acquiring an adaptive Lac^+^ mutation, a point which has been supported experimentally by evidence that Lac^+^ colonies with secondary mutations are mostly pure, not mixed for those mutations [29],[30], indicating that they generate the secondary mutations before, not after, becoming Lac^+^. In Text S1, we model a single HMS generating all mutants—single, double, triple, etc.—and ceasing hypermutability upon acquisition of a Lac^+^ mutation. Our model both predicts the apparent lower mutability of single Lac^+^ mutants seen previously [29],[30] and is compatible with the data presented here that Lac^+^ single mutants and multiple mutants arise from a common population by a common mutation mechanism—not two different mutation mechanisms (one involving *dinB* amplification and one not) as suggested [32].

### A Model for the Origin of the HMS

**Figure 4:**
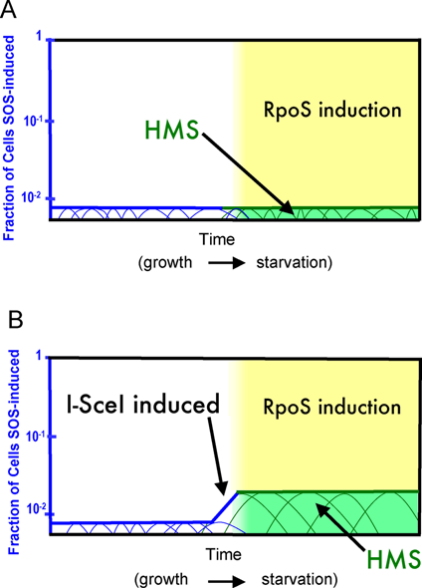
Model for the Differentiation of the HMS. (A) We suggest that differentiation of the HMS results from the convergence of three events: acquisition of a DNA double-strand break (DSB) or double-strand end (DSE, one end of a DSB); induction of the SOS DNA-damage response; and induction of the RpoS general stress-response (modified from Figure 5 in [2]). Spontaneous SOS induction occurs in about 1% (steady-state levels) of growing cells, about 60% of which were induced because of a DSB or DSE [28]. Individual cells may cycle in and out of the steady-state SOS-induced population, obtaining DNA damage, inducing SOS, then repairing the damage, and turning off SOS induction (rising and falling blue lines). Because repair of a DSB with SOS induction is not sufficient to cause mutagenesis—either stationary phase or induction of the RpoS response is also required [19]—we suggest that when the SOS-induced subpopulation is additionally induced for the RpoS stress response (yellow field), for example upon starvation, it becomes hypermutable: the HMS (green box). (B) Expectation for the HMS in experiments in which I-SceI-induced DSBs increased Lac^+^ mutagenesis. In these experiments (Tables 3,4 and Figure 3), I-SceI is induced from the P*_BAD_* promoter when the cells run out of glucose (stationary phase) and are plated onto lactose medium on which leaky expression from P*_BAD_* promotes I-SceI induction, DNA cleavage, and mutagenesis [19]. With stimulation of mutagenesis by I-SceI, Lac^+^ mutations remained coupled with chromosomal secondary mutations (Table 3, Figure 3E). This can be understood as depicted here: upon I-SceI induction, the fraction of cells with a DSB and an SOS response increases, causing an increase in the fraction of cells that will become the HMS when the RpoS response is induced upon starvation, and thus no decrease in the proportion of secondary mutations per Lac^+^ mutant (Table 3, Figure 3E). However, not all of the starved cells become HMS cells, in that most (Lac^−^ stressed cells) do not show the high genome-wide mutagenesis seen among Lac^+^ point mutants (Table 4), the descendents of the HMS. This might be because many cells receive no DSB, or because DSBs induced during starvation might induce SOS inefficiently.

The existence of a transiently mutable cell subpopulation indicates a differentiated state in a “bi-stable” cell population. We consider a possible model for the origin of the HMS (Figure 4A). We suggest that differentiation into an HMS cell will require three simultaneous events, all known to be required for HR-DSBR-dependent stress-induced mutagenesis in the Lac system: acquisition and repair of a DNA DSB [19]–[22]; induction of the SOS DNA-damage response [23]; and induction of the RpoS-controlled stationary-phase-, starvation- or general-stress response [10],[11]. The two stress responses transcriptionally upregulate DinB error-prone DNA polymerase 10-fold and ∼two-fold respectively [10],[27], which might be why they are required for stress-induced Lac point mutation [11],[23],[24], but this has not been demonstrated. The first two events—double-strand breakage and SOS induction—are probably related; that is, SOS might be induced by the requisite DSB. By contrast, in simple models (Figure 4A), induction of the RpoS response is imagined to occur independently of DSBs and SOS, based on different environmental inputs. That is, cells would have to sense at least two different deleterious conditions: DNA damage and an RpoS-inducing stress—while carrying a DSB—to differentiate into an HMS cell. A recent study from our laboratory showed that the SOS response is induced spontaneously in about 1% of growing cells, about 60% of that due to DSBs or double-strand ends (DSEs, half a DSB) [28]. We suggest that DNA damage provides the first stress-input sensed by the SOS response. We suggest that some of these SOS-induced cells are induced to levels of this graded response [39] appropriate for entry into the HMS at a later time if the RpoS response is induced. RpoS regulates a switch from high-fidelity to error-prone (mutagenic) DSBR mediated by Pol IV [19]. Thus, we propose that the HMS is differentiated by the convergence of these two stress-responses and a DSB/DSE in the observed [28] small subpopulation of cells, as illustrated in Figure 4A [2].

This model predicts that cells will spend differing lengths of time in the HMS. Pennington and Rosenberg [28] found that spontaneously SOS-induced cells, which induced GFP when SOS-induced, spent vastly different lengths of time in that condition. Upon recovery of the SOS-induced cells using fluorescence activated cell sorting (FACS), they found that some apparently repaired or ameliorated whatever DNA damage caused the response, then returned to cell cycling, proliferation, and formed colonies. Others stayed alive for at least eight hours after FACS but were unable to proliferate and form colonies for several days (i.e., did not end their SOS response and resume cell cycling). Friedman et al. also described the basis of the graded SOS response as a temporal gradation in how long individual cells remained induced (transcribing an SOS-GFP reporter gene) [39]. Thus, it seems likely that individual cells might spend varying lengths of time with SOS induced after DNA damage, and would thus, according to our model, spend very different lengths of time in the HMS. Cells would cycle in when they are SOS induced, and concurrently RpoS induced, then cycle out when either stress-response turns off. The SOS response is expected to be turned off when the DNA damage that instigated it is repaired. The RpoS response should turn off if the cells acquire an adaptive (e.g., Lac^+^) mutation that allows growth, and relief of their nutritional stress.

### Effects of Induced DSBs on HMS Size and Mutagenicity

According to this model, the I-SceI-mediated DSBs given here might be expected to increase the number of cells in the HMS (Figure 4B). In the experiments shown in Figure 3, P*_BAD_*I-*Sce*I transcription was repressed by glucose in the medium until stationary phase, when glucose would be exhausted and leaky expression from P*_BAD_* would ensue, just prior to plating on the selective lactose medium. Leaky expression from P*_BAD_*I-*Sce*I continues on the lactose selection pates [19]. We do not know what fraction of cells induce I-SceI under these conditions [19], nor how efficiently SOS is induced by I-SceI during stationary phase. However, our results indicate that not all cells become HMS cells as a result of I-SceI-mediated cleavage in these experiments. That is, the Lac^−^ stressed-cell population did not experience the same level of secondary mutagenesis as the I-SceI-induced point mutants (Table 4). This could be either because many cells did not receive an I-SceI-mediated DSB or because many of those that did failed to induce the SOS response. Although SOS-induction by I-SceI-mediated DSBs is efficient in growing cells [28], it is not known whether this is true in starving cells.

I-SceI-generated DSBs caused a small but statistically significant increase in the frequency of secondary mutations among Lac^+^ point mutants (Figure 3E, Table 3). This suggests a small increase in mutability of cells within the HMS and is not exclusive of the possible proposed increased in HMS population size (above, diagrammed Figure 4B). It is likely that the I-SceI-generated DSBs are repaired using a sister DNA molecule, which would itself carry the I-SceI cutsite. This would cause multiple rounds of I-SceI-mediated DNA cleavage, and, we suggest, prolonged induction of the SOS response, potentially causing cells to stay longer in the HMS condition, accumulating more mutations genome-wide.

### Mutability of the HMS and Adaptation at the Cell and Population Levels

Although an HMS can produce adaptive mutations, neutral and deleterious mutations will also be produced. Can an HMS enhance fitness? We suggest here that differentiation of an HMS may enhance fitness of individual cells in it, but also, separately, of the larger population.

Based on findings presented in this study, we estimated that in addition to the selected Lac^+^ mutation, cells that underwent stress-induced mutagenesis would carry between about one and 3.4 mutation clusters (of one or more mutations) per genome (Results). We also supported previous findings that mutations at *lac* occur in clusters [35] and estimated the number of mutations per cluster to be about 1.67 (Text S1). If the genome-wide mutations also occur in clusters, as mutations at *lac* do [35], this would then predict a frequency of between two and six mutations in addition to Lac^+^ per genome (1 to 3.4 mutations×1.67 mutations per cluster). This is a maximal estimate given that chromosomal mutations might not be clustered similarly, though this hypothesis seems unlikely. Could a developmental program that generates at most 2–6 additional mutations per genome be adaptive for the rare cells that generate an adaptive mutation? This will depend on how many of the additional mutations are not synonymous, and how many of the genes they fall in are relevant to the specific environment the stressed bacterium inhabits. We have no way to assess the latter, but our rough estimate of the former is that about 29.5% of all mutations falling in anywhere in the genome will affect coding (Text S1). Even if every gene mattered for fitness in the bacterium's particular environment—an unlikely prospect—this would mean that on the low end of the estimate for additional mutations (two) the probability of a non-neutral, additional mutation is 1−(1−0.295)^2^ = 0.503, such that 50% of the Lac^+^ adaptive mutants would not be harmed by having been through the hypermutable state. On the high end, the probability of a non-neutral, additional mutation 1−(1−0.295)^6^ = 0.877 (for 6 additional mutations), but this too is probably significantly reduced by the likelihood that many of the genes in the genome are irrelevant to fitness in any given environment (supported by a recent study [40]). The evolutionarily conserved *E. coli* core genome is only about half of the genes [41], such that it is possible that many of the rest are dispensable in at least some circumstances. At this gross level, it appears plausible that adaptive mutants could be generated without undue burden of coincident maladaptive mutations.

As a nonexclusive alternative, we suggest that HMS cells could produce adaptive and non-adaptive mutations and then sometimes mix their genomes with those of others in the clone, and so enhance populational fitness. A low rate of genetic mixing can allow individual mutations to be selected independently of their genetic background, thus increasing the probability of fixation of adaptive mutations [42] while lowering the probability of fixation of deleterious mutations [43], altogether benefiting the population. The mixing could occur via horizontal transmission for example by conjugation, phage-mediated transduction and natural transformation. Notably, all of these transmission modes are stimulated by stress. Conjugation is promoted by starvation stress (e.g., [44]). Induction of some prophages from the lysogenic state (and so potentially the ability to act as a transductional donor) is activated by the SOS DNA-damage stress response (e.g., [45]). Natural competence is induced by starvation and is controlled in *Bacillus subtilis* by the same Com gene regulators that also activate a *B. subtilis* stress-induced mutagenesis program [46]. Perhaps stress provokes both differentiation of an HMS while simultaneously inducing the programs that promote genetic mixing. The HMS cells could act as either donors or recipients. As donors, HMS cells could act as “mutation factories” that export mutations to other cells in the clone. As recipients, HMS cells could potentially lose deleterious mutations by genetic mixing.

### Evolvability and the Regulation of Mutagenesis in Time, Space, and a Cell Subpopulation

Mechanisms of stress-inducible mutagenesis in bacteria, yeast, and human cells appear to limit the dangerous experiment of mutagenizing a genome in at least three important ways, each adding a layer of regulation: in time, specifically to times of stress; in genomic space to localized genome regions; and to a cell subpopulation ([19] reviewed [2]). The first two are now well documented in many different organisms and circumstances (reviewed below) and the third, so far, is demonstrated only in two circumstances of bacterial mutation. All three strategies may enhance inherent “evolvability” of cells and organisms that employ them [2],[19],[47],[48].

First, the coupling of mutagenesis mechanisms/programs to cellular stress responses limits mutagenesis to times of stress, when cells/organisms are maladapted to their environments. The bacterial RpoS-controlled general-, starvation-, or stationary-phase-stress response, positively regulates many mutagenic processes: the fidelity of DSBR, promoting point mutagenesis during stress in *E. coli* [19]; stress-induced mutagenesis in aging colonies of an *E. coli* natural isolate [6]; stress-induced few-base deletions in *Pseudomonas putida* [49]; and genome rearrangements such as stress-induced *lac*-amplification [11]; phage Mu-transposon mediated deletions in *E. coli* [50],[51]; and starvation-promoted transpositions in *P. putida* [52], among others [2]. The diversity of these processes, and the fact that even among point-mutation pathways at least two different DNA polymerases are involved (DinB for Lac [19],[24] and *P. putida* [53] and Pol II for mutagenesis in aging colonies [6]), suggests that RpoS promotes genome instability by more than one mechanism. The competence (natural-transformation) stress response to starvation in *B. subtilis* is required for starvation-stress-induced mutagenesis in that organism [46]. Two different human stress responses to hypoxia transcriptionally down-regulate mismatch-repair proteins, causing increased genome instability [54]–[57], and transcriptionally down-regulate BRCA1 and RAD51 homologous-recombinational (HR-) DSB-repair proteins, potentially promoting genome rearrangements in response to hypoxic stress [58]–[60]. The SOS DNA-damage response is the classic stress response that promotes mutagenesis both at sites of DNA damage and elsewhere [reviewed, 61] including in many stress-induced mutagenesis pathways in various bacteria [reviewed, 2]. Similarly a eukaryotic DNA-damage response to shortened telomeres promotes transposition in yeast [62]. All of these stress-response-controlled mutation mechanisms promote genetic change specifically when cells/organisms are maladapted to their environments, i.e., are stressed, potentially accelerating evolution specifically then. They are varied and suggest multiple independent evolutions of this strategy.

Second, in many systems, mutagenesis is limited in genomic space to small genomic regions. This may also be evolutionarily advantageous in potentially limiting accumulation of deleterious mutations in rare adaptive mutants, as well as promoting concerted evolution within linked genes and gene families [2],[19],[47],[48]. Restriction of mutagenesis in genomic space is evident in the coupling of both stress-induced point mutagenesis and gene amplification/genome rearrangement to acts of HR-DSBR in the Lac system [19]; and DSB-repair associated mutations in yeast [63], and is implied in *E. coli* ciprofloxacin-induced resistance mutations [64], Salmonella bile-induced resistance mutations [65],[66], and yeast stress-induced mutations [67], all of which require DSB-repair proteins and so may occur during localized DSBR. Similarly, the potential genome instability in human cells caused by a switch to non-homologous DSBR is suggested by down-regulation of human homologous-DSBR genes during stress and could potentially also localize mutagenesis [59],[60]. The association of transcription with mutagenesis also implies mutational localization in the genome in stressed in *E. coli* [68], yeast [69], and this association is also implied in *B. subtilis* [70] and in more indirect *E. coli* data [71]. Mutational clustering is observed generally in many organisms [72], including mouse [73], and also in somatic hypermutation of immunoglobulin genes [74]. Thus, many systems display localization of mutagenesis in genomic space, a potentially adaptive strategy [2],[19],[47],[48].

Finally in the *E. coli* Lac system, we see a third layer of limitation/regulation of mutagenesis: its restriction to a small cell subpopulation [29],[30],[31, and here]. This strategy may further buffer populations against the deleterious effects of mutagenesis by exposing only a minority of the members to these effects. Though dangerous to individual organisms, this differentiation of a bi-stable population can be advantageous to the clone, allowing the population to hedge its bets should stress be relieved suddenly [75]. Moreover differentiation of a HMS could allow some cells to both generate mutations and mix their genomes with others in the clone, as discussed in the previous section, reducing the risk of deleterious-mutation load. Competence for natural transformation in *B. subtilis*, which promotes genetic diversity by recombination, similarly engages only a subpopulation of stressed bacterial cells, as does sporulation [75]. How general the HMS strategy may be is not known. One other mechanism of mutagenesis in *E. coli* has shown evidence of engaging a HMS: stress-induced Trp reversions [76], which did not require HR-DSBR proteins, and so occurred by a mechanism different from the HR-DSBR-associated stress-induced point mutagenesis studied here. A report of mutation “showers” in mouse somatic cells [73] suggests bouts of localized transient mutability, which might be limited to a HMS, but this has not been investigated. Given the prevalence of bi-stable (subpopulation) states in bacteria [75] and the ability of all organisms to differentiate, the possible generality of HMS strategies seems likely.

## Materials and Methods

### *E. coli* Strains and Mutation Assays

*E. coli* strains used are shown in Table 5. Stress-induced mutation assays were performed as described [21] with two exceptions. First, the M9 glycerol medium in which cells are grown prior to plating on lactose medium was supplemented with 0.001% glucose to repress P*_BAD_*, controlling the I-SceI endonuclease, as were LBH rifampicin plates onto which cfu were spread for daily viable cell measurements. Second, in order to be able to recover Lac^+^ mutants carrying secondary auxotrophic mutations, the usual minimal lactose medium on which Lac^+^ mutants are selected was supplemented with the following additions that cannot be used as a carbon source [32],[77] at the following concentrations (mM): histidine, 0.1; isoleucine, 0.3; leucine, 0.3; lysine, 0.3; methionine, 0.3; phenylalanine, 0.3; threonine, 0.3; tryptophan, 0.1; tyrosine, 0.1; valine, 0.3; adenine hydrochloride, 0.5; guanine, 0.3; thymine, 0.32; and uracil, 0.1.

**Table 5:** *E. coli* Strains Used.

Strain	Relevant Genotype	Origin
FC29	Δ(*lac-proB*)_xiii_ *ara thi* [F' Δ*lacIZ proAB* ^+^]	[8]
FC36	Δ(*lac-proB*)_xiii_ *ara thi* Rif^R^	[8]
FC40	FC36 [F' *lacI33*Ω*lacZ proAB* ^+^]	[8]
SMR4562	FC36 [F' *lacI33*Ω*lacZ proAB* ^+^]	Independent construction of FC40 [23]
SMR6272	SMR4562 Δ*araBAD567*	[19]
SMR6276	SMR6272 Δ*att*λ::P*_BAD_*I-*Sce*I	[19]
SMR6277	SMR6272 Δ*att*λ::P*_BAD_*	[19]
SMR6280	SMR6272 Δ*att*λ::P*_BAD_*I-*Sce*I [F' *mhpA32*::miniTn*7*Kan(I-SceI site)]	[19]
SMR6281	SMR6272 Δ*att*λ::P*_BAD_* [F' *mhpA32*::miniTn*7*Kan(I-SceI site)]	[19]

Unselected secondary mutations among Lac^+^ mutants were assayed by purifying Lac^+^ point mutants from days 4 and 5 on LBH plates containing 1% glucose (Glu), 100 µg/ml rifampicin (Rif), 40 µg/ml 5-bromo-4-chloro-3-indoyl β-D-galactoside (X-gal), (glucose for repression of P*_BAD_*, Rif to exclude FC29 scavenger cells, and X-gal to screen out *lac*-amplified clones per [14]. These plates were incubated overnight at 37°C. Isolated colonies were patched onto grids on the same medium (LBH Rif X-gal Glu plates) for replica plating and incubated overnight at 37°C. These master plates were replica-plated (printed) via velvets to M9 vitamin B1 minimal glucose (0.1%) plates to screen for auxotrophs; M9 B1 minimal glucose 5-fluorocytosine (5-FC, 50 µg/ml) plates to screen for 5-FC resistance (caused by mutation in the F'-borne *codAB* genes, per [29], confirmed by sensitivity to 5-fluorouracil) and MacConkey maltose and MacConkey xylose pH indicator plates to screen for defects in maltose and xylose fermentation (per [29]). Mutants were confirmed by purifying from the LBH-Rif-X-gal-Glu master plate and retesting on the appropriate selective or indicator medium. Unselected secondary mutations in Lac^−^ unstressed cells were assayed by plating aliquots on LBH-Rif-X-gal-Glu plates, incubating overnight at 37°C, followed by patching isolated colonies onto the same medium, and treating as above. Similarly, unselected secondary mutations in Lac^−^ starved cells were assayed by taking plugs of agar from between visible colonies at day 3 of incubation (comparable to day-5 Lac^+^ colonies due to the 2-day colony-formation time) on M9 B1 lactose plates, with supplements as above, and suspending in M9 buffer. Aliquots were plated on LBH-Rif-X-gal-Glu medium, incubated overnight at 37°C, and isolated colonies were patched, grown and replica plated as described above.

### Induction of Lac^+^ Mutagenesis by I-SceI

To increase Lac^+^ mutant frequency in the Lac assay, we employed the chromosomal *E. coli* I-SceI endonuclease system constructed by our lab [78] and used by us and others [e.g.],[19],[79,80]. I-SceI endonuclease makes a specific DSB at an 18bp cutsite, not normally present in the *E. coli* genome [81]. In this construct [78], the I-*Sce*I-endonuclease open reading frame is cloned in front of the *E. coli* arabinose-inducible P*_BAD_* promoter and the expression cassette is present in the *E. coli* chromosome, replacing the phage lambda attachment site, *att*λ. We used strains carrying a chromosomal cassette of the P*_BAD_* promoter with or without the I-*Sce*I gene and strains with or without the I-SceI cutsite on the F' episome, 4.5 kb from of the *lac* allele in the *mhpA* (EG20273) gene [19] (Table 5).

### Sequencing

The *lac* region of Lac^+^ mutants containing chromosomal secondary mutations was PCR amplified with primers 5′-ATATCCCGCCGTTAACCACC-3′ and 5′-CGGAGAAGCGATAATGCGGTCGA-3′ and sequenced (Lone Star Labs Inc., Houston, TX) with primer 5′-ATATCCCGCCGTTAACCACC-3′.

## Supporting Information

Table S1 Similar Secondary Mutation Frequencies in New and Old Strains.
(0.06 MB DOC)

Table S2 Summary of Generation-Dependent Lac^+^ Reversion-Mutation Sequences.
(0.06 MB DOC)

Text S1 Supplementary Text and References.
(0.10 MB DOC)
