# Supplementary material for: Mutability and Importance of a Hypermutable Cell Subpopulation that Produces Stress-Induced Mutants in Escherichia coli
Source: PLoS Genet. 2008 Oct 3;4(10):e1000208. doi: 10.1371/journal.pgen.1000208 (PMC2543114; doi:10.1371/journal.pgen.1000208)
Supplement: Table S1 — Similar Secondary Mutation Frequencies in New and Old Strains. (0.06 MB DOC) [file pgen.1000208.s001.doc]

**Table S1.** Similar Secondary Mutation Frequencies In New and Old Strainsa

|  |  | Secondary Mutant Frequency Among Lac^+^ Mutants | |
| --- | --- | --- | --- |
| Replicon carrying the unselected mutation | Mutant Phenotype | SMR6277^b^  (New Strain) | FC40^c^  (Old Strain) |
| F’ | 5-FC^R^ | 62/10,867 (5.7 x 10^-3^) | 121/19,647 (6.2 x 10^-3^) |
| Chromosome | Mal^-^ | 7/10,867 | 31/42,617 |
|  | Xyl^-^ | 4/10,867 | 22/42,617 |
|  | Total | 11/10,867 (1.0 x 10^-3^)* | 53/42,617 (1.2 x 10^-3^)* |

^a^ New and old strains were assayed by the method of direct transfer by replica plating of Torkelson *et al.* [29]. The similar frequencies when new and old strains are assayed by the same method indicate that the higher frequencies reported here (Tables 1 - 3) with the new strain using the purify-and-patch method reflect the greater sensitivity of the purify-and-patch method, rather than strain differences.

^b^ Strain SMR6277, the “P*_BAD_* Only” strain is isogenic with FC40 except that the phage lambda attachment site, *att*λ, is replaced with the P*_BAD_* promoter: ∆*att*λ::P*_BAD_*. This is a negative-control strain, that makes no I-SceI-endonuclease-induced DNA double-strand breaks (DSBs), for the strain with ∆*att*λ::P*_BAD_*I-*Sce*I and an I-SceI cutsite, which makes DSBs [19].

^c^ Strain FC40. Data from [29].

* No significant difference (*p* = 0.697, z-test with Yates correction). This indicates that ∆*att*λ::P*_BAD_* did not influence mutant frequency measurements.
