# Supplementary material for: Mutability and Importance of a Hypermutable Cell Subpopulation that Produces Stress-Induced Mutants in Escherichia coli
Source: PLoS Genet. 2008 Oct 3;4(10):e1000208. doi: 10.1371/journal.pgen.1000208 (PMC2543114; doi:10.1371/journal.pgen.1000208)
Supplement: Table S2 — Summary of Generation-Dependent Lac+ Reversion-Mutation Sequences. (0.06 MB DOC) [file pgen.1000208.s002.doc]

| Kind of Mutation |  | Number Observed | Percent of Total |
| --- | --- | --- | --- |
| -1 at mononucleotide repeat ^b^ |  | 23 | 53 |
| -1 not at mononucleotide repeat |  | 3 | 7 |
| +2 |  | 3 | 7 |
| +5 |  | 1 | 2.3 |
| +8 |  | 1 | 2.3 |
| -2^b^ |  | 1 | 2.3 |
| Large deletion^c^ |  | 6 | 14 |
| Large insertion^d^ |  | 5 | 12 |
|  |  |  |  |
| Total |  | 43 | 100 |

**Table S2.** Summary of Generation-Dependent Lac^+^ Reversion-Mutation Sequences^a^

^a^ Summary of generation-dependent (Day-2) Lac^+^ reversion mutation sequences from [12, 13].

^b^ This number includes one complex mutant from [13] that carried a -1 deletion in a mononucleotide repeat that corrected the reading frame, and an additional -2 deletion that would not affect coding because it was outside of the region in which deletion or addition mutations could compensate for the -1 deletion.

^c^ The large deletion of [13] was -110 bp. The five large deletions of [12] were large enough to shift the bands of PCR products on gels.

^d^ The large insertion of [13] was a 67 bp duplication. The four large deletions of [12] were large enough to shift the bands of PCR products on gels.
