# Supplementary material for: Mutability and Importance of a Hypermutable Cell Subpopulation that Produces Stress-Induced Mutants in Escherichia coli
Source: PLoS Genet. 2008 Oct 3;4(10):e1000208. doi: 10.1371/journal.pgen.1000208 (PMC2543114; doi:10.1371/journal.pgen.1000208)
Supplement: Text S1 — Supplementary Text and References. (0.10 MB DOC) [file pgen.1000208.s003.doc]

**Supporting Text**

**Gonzalez C. *et al.,* *PLoS Genetics* 2008**

**Estimate of mutations per mutation cluster**

Mutations in the Lac system appear to be clustered locally in the DNA [1]. We can make a rough estimate of the number of mutations per mutation cluster from data on the apparent clustering of Lac^+^ mutants with the linked mutations in the *codAB* genes, 10kb from *lac*. Loss-of-function mutations in the *codAB* genes, which conferred resistance to the nucleotide analogue 5-fluorocytosine (5-FC^R^) (but not also to 5-fluorouracil, as chromosomal *upp* mutations do), were shown not to form independently of Lac^+^ mutations, whereas unlinked chromosomal mutations did [1]. Additionally in our study here, 5-FC^R^ mutations in *codAB* were more frequent among Lac^+^ mutants than were unlinked mutations (Table 3, first two columns). The previous data [1] imply that *codAB* mutations cluster with *lac* mutations which, subsequent work indicates, are initiated by double-strand-end formation at the transfer origin, *oriT*, of the F’ plasmid, about 75kb away [2]. In that study [2], the TraI endonuclease that nicks the transfer origin was shown to be required for stress-induced mutagenesis but was not required if I-SceI-mediated DSBs were made near *lac*, and the I-SceI-generated DSBs promoted mutation more efficiently (shown also here in Figure 3A-D: 70-fold higher mutagenesis promoted by I-SceI than the wild-type, TraI-dependent background). This suggests a cluster size of at least 75kb. We observed 26/3437 (7.6 x 10^-3^) *codAB* mutations linked to the *lac* gene (Table 3, data from “P*_BAD_*-only” + “enzyme-only” strains). Given an estimated mutation-target size of 848bp (“Basepairs targeted in 5-FC^R^ mutant screens”, below) this implies 8.9 x 10^-6^ mutations per bp in a cluster region. Assuming a cluster size of 75kb, we calculate: (8.9 x 10^-6^ mutations per bp) (75,000 bp) resulting in at least 0.67 mutations in addition to Lac^+^ mutation already present, bringing the total to 1.67 mutations per cluster.

**Basepairs targeted in auxotrophic-mutant screen, this study**

Medium with the supplements listed in Materials and Methods (Mutation Assay) could support the growth of cells with loss-of-function mutations in the following genes of the following sizes (obtained from Colibri <http://genolist.pasteur.fr/Colibri/>): adenine and guanine (*purF* (EG10794) 1518bp, *purD* (EG10792) 1290bp, *purL* (EG10797) 3888bp, *purM* (EG10798) 1038bp, *purK* (EG10796) 1068bp, *purE* (EG10793) 510bp, *purC* (EG10791) 714bp, *purB* (EG11314) 1371bp, *purH* (EG10795) 1590bp, *purA* (EG10790) 1299bp, *guaA* (EG10420) 1578bp and *guaB* (EG10421) 1467bp [3]); uracil and thymine (*pyrB* (EG10805) 936bp *pyrI* (EG10811) 462bp, *pyrC* (EG10806) 1047bp, *pyrD* (EG10807) 1011bp, *pyrE* (EG10808) 642bp, *pyrF* (EG10809) 738bp, and *thyA* (EG11002) 795bp [4]); histidine (*hisA* (EG10444) 738bp, *hisB* (EG10445) 1068bp, *hisC* (EG10446) 1071bp, *hisD* (EG10447) 1305bp, *hisF* (EG10448) 777bp, *hisG* (EG10449) 900bp, *hisH* (EG10450) 591bp, and *hisI* (EG10451) 612bp [5]); threonine and lysine (*asd* (EG10088) 1104bp, *thrA* (EG10998) 2463bp, *metL* (EG10590) 2433 bp, *lysC* (EG10550) 1350bp, *dapA* (EG10205) 879bp, *dapB* (EG10206) 822bp, *argD* (EG10066) (*dapC*) (1221bp, *dapD* (EG10207) 825bp, *dapE* (EG10208) 1128bp, *dapF* (EG10209) 825bp, *lysA* (EG10549) 1263bp, *thrB* (EG10999) 933bp and *thrC* (EG11000) 1287bp [6]); methionine (*metA* (EG10581) 930bp, *metB* (EG10582) 1161bp, *metC* (EG10583) 1188bp, *metE* (EG10584) 2262bp, and *metH* (EG10587) 3684bp [7]); phenylalanine, tyrosine and tryptophan (*aroA* (EG10073) 1284 bp, *aroB* (EG10074) 1089bp, *aroC* (EG10075) 1086bp, *aroD* (EG10076) 759bp, *aroE* (EG10077) 819bp, *aroF* (EG10078) 1071bp, *aroG* (EG10079) 1053bp, *aroH* (EG10080) 1047bp, *aroK* (EG10081) 522bp, *aroL* (EG10082) 525bp, *pheA* (EG10707) 1161bp, *tyrA* (EG11039) 1122bp, *tyrB* (EG11040) 1194bp, *trpE* (EG11028) 1563bp, *trpA* (EG11024) 807bp, *trpB* (EG11025) 1194bp, *trpC* (EG11026) 1359bp, and *trpD* (EG11027) 1596bp [8]); leucine, isoleucine and valine (*ilvA* (EG111027) 1545bp, *ilvC* (EG10495) 1476bp, *ilvD* (EG10496) 1849bp, *ilvE* (EG10497) 930bp, *avtA* (EG10107) 1254bp, *leuA* (EG11226) 1572bp, *leuB* (EG11577) 1095bp, *leuC* (EG11576) 1401bp, and *leuD* (EG11575) 606bp [9]). These 72 loci encompass 86,761 bp of DNA, one third [10] of which (28,920) are predicted to produce loss-of-function phenotypes if mutated.

Although this one third [10] is based on substitution mutations that alter protein coding, and at least some of the mutations in the Lac system are frameshift mutations (those that revert the *lac* +1 frameshift allele), our unpublished data indicate that most chromosomal secondary mutations in one gene in a loss-of-function mutation assay are substitutions (JF Petrosino, R Galhardo, LD Morales, SM Rosenberg, unpublished data). Thus, the one-third approximation appears reasonable.

**Basepairs targeted in Mal^-^ mutant screens**

Of the thirteen mal genes, six confer a Mal^-^ phenotype [11,12]: *malT* (EG10562) 2706bp, *malE* (EG10554) 1191bp, *malF* (EG10555) 1545bp, *malG* (EG10556) 891bp, *malK* (EG10558) 1116bp and *malQ* (EG10561) 2085bp [12] (sizes from Colibri <http://genolist.pasteur.fr/Colibri/>). This totals 3178bp in the genome; considering the Colibri annotated size of these 6 genes, we have 9534 total bp, 1/3 of which [10] are expected to cause loss-of-function if mutated: 3178.

**Basepairs targeted in Xyl^-^ mutant screens**

Loss of function of four genes of the following sizes can give rise to Xyl^-^ phenotype [11]: *xylA* (EG11074) 1323bp; *xylB* (EG11075) 1455bp; *xylE* (EG11076) 1476bp; and *xylR* (EG20253) 1179 (sizes from Colibri <http://genolist.pasteur.fr/Colibri/>). This totals 5433bp, one third [10] of which (1811) are predicted to cause loss of function if mutated.

**Basepairs targeted in 5-FC^R^ mutant screens**

One third of basepairs [10] in the *codA* (1284bp) and *codB* (1260bp) genes, loss of function of which confers resistance to 5-fluorocytosine, and not simultaneously to 5-fluorouracil [4,13,14], comprises an 848bp mutation target. Sizes from Colibri <http://genolist.pasteur.fr/Colibri/>.

**Estimate of probability of any mutation falling in the *E. coli* genome being neutral**

The *E. coli* genome is 4,639,221 bp, 87.8% of which is protein encoding for 4,073,236 bp of DNA in protein-encoding genes. (A further 0.8% specifies stable, non-message RNAs [15], not considered further here because it is a small fraction of the total.) If we assume, first, that only DNA in protein-encoding genes contributes to phenotype changes if mutated, and, second, that changes to only 1/3 of the bp in the protein-encoding-gene DNA will be non-neutral [10], then changes to any of 1,370,250 bp of DNA out of the total 4,639,221 bp should cause a phenotype. That is, 1,357,745 / 4,639,675 = 29.3% of all mutations should be non-neutral.

**A single HMS can generate all Lac^+^ stress-induced mutants**

A previous study examined the distribution of Lac^+^ stress-induced mutants carrying zero, one, two or more additional, phenotypically detected secondary mutations throughout their genomes [16]. They reported a higher mutation rate among Lac^+^ mutants carrying known secondary mutations than those Lac^+^ mutant with no known secondary mutation in their study and in data from a previous study [17]. The authors suggested that this might mean that most of the Lac^+^ single mutants descended from a different population of cells, with a different and lower mutation rate, than the hypermutable cell subpopulation (HMS) that generated double, triple and multiple mutants. That is, there seemed to be too many Lac^+^ single mutants. Here we show that if one allows for the exit of cells from a HMS upon acquisition of a beneficial (Lac^+^) mutation, then a single HMS is capable of producing all of the mutants (single and multiple).

If we assume that the occurrence of mutations is a Poisson process and that new mutations are independent of ones that have already occurred, then, if mutagenesis were *not* shut off after becoming Lac^+^, the likelihood of having exactly one mutation that would be beneficial (in the current case, a mutation that would result in the cell becoming Lac^+^) is

*P1* = m·e^-m^ · *a*

where m is the average number of mutations per genome including the mutation to Lac^+^ (Discussion, “Mutability of the HMS and Adaptation at the Cell and Population Levels” 2-6, plus one for Lac^+^), and *a* is the frequency of advantageous mutations that allow the cell to become unstressed out of all the mutations that have a phenotypic effect.

If we assume that individuals that become Lac^+^ cease being hypermutable, then the probability of having exactly one beneficial mutation is simply the probability of having a good mutation on the first step, and that the first step has indeed occurred in the relevant time frame. That is,

*P2* = (1 – e^- m^) · *a*

*P2*>*P1* for any value of m>0, meaning that, if Lac^+^ mutants leave the HMS, there will be more Lac^+^ mutants with no other mutation than there would be if Lac^+^ mutants did not leave the HMS upon acquiring the Lac^+^ mutation. The extent of this increase can be calculated from the formulae above. Given that m is between 3 and 7 (including the Lac^+^ mutation, Discussion, “Mutability of the HMS and Adaptation at the Cell and Population Levels”) and that *a* is the number of base positions at which a mutation can revert Lac^-^ to Lac^+^ relative to the length of bp that would cause a phenotype (131/1.36 x 10^6^ basepairs), *a* = 9.6 x 10^-5^. With m = 3, *P1* is 1.43 x 10^-5^ and *P2* is 9.1 x 10^-5^. Thus Lac^+^ with no other mutation is 6.4-fold more probable on the hypothesis of leaving the HMS than on the simple hypothesis of remaining hypermutable. If m = 7, the ratio of *P1* to *P2* is about 160, that is, we expect ~160-fold more Lac^+^ single mutants if cells leave the HMS upon acquiring a Lac^+^ mutation.

**Reconciliation with previous modeling**

Roth et al. [18] modeled the parameters of adaptive mutation in the Lac system. Among models that they considered was the model used here that allowed exit from the HMS upon reverting to Lac^+^, and included the possibility that starved cells can enter the HMS during the experiment, thus maintaining a constant size of HMS. Their modeling led them to conclude that transient mutagenesis would be unlikely to provide an evolutionary advantage. They gave three reasons for this conclusion. First, it was necessary to postulate a mutation rate increase of 10^4^ to 10^5^ in the HMS to explain the observed yield of Lac^+^ cells, which seemed to them too high given that other data had led them to estimate a 35-fold increase. However the other data compared mutations among Lac^+^ cells with those found in the stressed Lac^-^ cells in one study [16], and with Lac^+^ colonies arising on day two in the other study [19]. The stressed cells already show an enhanced mutation rate [20]. Similarly, day-two Lac^+^ colonies include not only generation-dependent mutants, but also many stress-induced mutants, and so also have enhanced mutation rate [e.g., 21]. This means that the estimated 35-fold increase in mutation rate in the HMS should have been higher because both “control” comparison populations were already engaged in stressed-induced mutagenesis.

Second, modeling suggested that Lac^+^ mutants would carry, on average, 8 non-lethal secondary mutations [18]. This corresponds well with the number of 2 to 6 mutations in addition to *lac* obtained empirically in this study. Third, the modeling revealed an expected increase in mutation of 85-fold among Lac^-^ stressed cells [18] as compared with a published value of 4-fold increase, above the level of spontaneous mutation, in the overall yield of unselected mutations in Lac^-^ stressed cells [20]. The calculated expectation of 85-fold increase in mutation in the Lac^-^ stressed cells might be too high because the mutation rate of a gene in the F plasmid is about 20-fold higher than the same gene in the chromosome [20,22] such that the overall chromosomal mutation load should be proportionately less. From these considerations we conclude that the modeling of Roth et al. [18] does not demonstrate the improbability of stress-induced increase in mutability, but rather supports the conclusions above.

**References**

1. Bull HJ, McKenzie GJ, Hastings PJ, Rosenberg SM (2000) Evidence that stationary-phase hypermutation in the *E. coli* chromosome is promoted by recombination. Genetics 154: 1427-1437.

2. Ponder RG, Fonville NC, Rosenberg SM (2005) A switch from high-fidelity to error-prone DNA double-strand-break repair underlies stress-induced mutation. Mol Cell 19: 791-804.

3. Zalkin H, Nygaard P (1996) Biosynthesis of purine nucleotides. In: Neidhardt FC, Curtiss III R, Ingraham JL, Lin ECC, K.B. L et al., editors. *Escherichia coli* and *Salmonella* Cellular and Molecular Biology. Second ed. Washington, D.C. : ASM Press. pp. 561-579.

4. Neuhard J, Kelln RA (1996) Biosynthesis and conversions of pyrimidines. In: Neidhardt FC, Curtiss III R, Ingraham JL, Lin ECC, Low KB et al., editors. *Escherichia coli* and *Salmonella* Cellular and Molecular Biology. second ed. Washington, D. C.: ASM Press. pp. 580-599.

5. Winkler ME (1996) Biosynthesis of histidine. In: Neidhardt FC, Curtiss III R, Ingraham JL, Lin ECC, K.B. L et al., editors. *Escherichia coli* and *Salmonella* Cellular and Molecular Biology. Second ed. Washington, D.C. : ASM Press. pp. 485-505.

6. Patte J-C (1996) Biosynthesis of threonine and lysine. In: Neidhardt FC, Curtiss III R, Ingraham JL, Lin ECC, K.B. L et al., editors. *Escherichia coli* and *Salmonella* Cellular and Molecular Biology. Second ed. Washington, D.C. : ASM Press. pp. 528-541.

7. Greene RC (1996) Biosythesis of methionine. In: Neidhardt FC, Curtiss III R, Ingraham JL, Lin ECC, K.B. L et al., editors. *Escherichia coli* and *Salmonella* Cellular and Molecular Biology. Second ed. Washington, D.C. : ASM Press. pp. 542-560.

8. Pittard AJ (1996) Biosynthesis of the aromatic amino acids. . In: Neidhardt FC, Curtiss III R, Ingraham JL, Lin ECC, K.B. L et al., editors. *Escherichia coli and Salmonella* Cellular and Molecular Biology. Second ed. Washington, D.C. : ASM Press. pp. 458-484.

9. Umbarger HE (1996) Biosynthesis of the branched chain amino acids. In: Neidhardt FC, Curtiss III R, Ingraham JL, Lin ECC, K.B. L et al., editors. *Escherichia coli* and *Salmonella* Cellular and Molecular Biology. Second ed. Washington, D.C. : ASM Press. pp. 442-457.

10. Drake JW, Charlesworth B, Charlesworth D, Crow JF (1998) Rates of spontaneous mutation. Genetics 148: 1667-1686.

11. Lin ECC (1996) Dissimilatory pathways for sugars, polyols, and carboxylates. In: Neidhardt FC, Curtiss III R, Ingraham JL, Lin ECC, K.B. L et al., editors. *Escherichia coli* and *Salmonella* Cellular and Molecular Biology. Washington, D.C. : ASM Press. pp. 307.

12. Boos W, Shuman H (1998) Maltose/maltodextrin system of Escherichia coli: transport, metabolism, and regulation. Microbiol Mol Biol Rev 62: 204-229.

13. De Haan PG, Felix HS, Peters RC (1972) Mapping of the gene for cytosine deaminase on the *Escherichia coli* chromosome. Antonie van Leevwenhoek 38: 257-263.

14. Lind RM, Sukhodolets VV, Smirnov YU (1973) Mutations affecting deamination and transport of cytosine in *Escherichia coli*. Genetika 9: 116-121.

15. Blattner FR, Plunkett III G, Bloch CA, Perna NT, Burland V, et al. (1997) The complete genome sequence of *Escherichia coli* K-12. Science 277: 1453-1474.

16. Rosche WA, Foster PL (1999) The role of transient hypermutators in adaptive mutation in *Escherichia coli*. Proc Natl Acad Sci USA 96: 6862-6867.

17. Torkelson J, Harris RS, Lombardo M-J, Nagendran J, Thulin C, et al. (1997) Genome-wide hypermutation in a subpopulation of stationary-phase cells underlies recombination-dependent adaptive mutation. EMBO J 16: 3303-3311.

18. Roth JR, Kofoid E, Roth FP, Berg OG, Seger J, et al. (2003) Regulating general mutation rates. Examination of the hypermutable state model for Cairnsian adaptive mutation. Genetics 163: 1483-1496.

19. Slechta ES, Harold J, Andersson DI, Roth JR (2002) The effect of genomic position on reversion of a lac frameshift mutation (*lacIZ33*) during non-lethal selection (adaptive mutation). Mol Microbiol 44: 1017-1032.

20. Bull HJ, Lombardo M-J, Rosenberg SM (2001) Stationary-phase mutation in the bacterial chromosome: recombination protein and DNA polymerase IV dependence. Proc Natl Acad Sci USA 98: 8334-8341.

21. Harris RS, Feng G, Ross KJ, Sidhu R, Thulin C, et al. (1999) Mismatch repair is diminished during stationary-phase mutation. Mutat Res 437: 51-60.

22. Foster PL (1997) Nonadaptive mutations occur in the F' episome during adaptive mutation conditions in *Escherichia coli*. J Bacteriol 179: 1550-1554.
